# The prevalence of type 2 diabetes mellitus and associated factors among adult patients attending Outpatient Department at Jinella Health Center, Harar, Ethiopia: A cross-sectional study

**DOI:** 10.1097/MD.0000000000045938

**Published:** 2025-11-14

**Authors:** Fasil Getu, Addisu Tesfaye, Birhanu Genanew, Abdikani Rashid, Muluken Walle

**Affiliations:** a Department of Medical Laboratory Science, Institute of Health Sciences, Jigjiga University, Jigjiga, Ethiopia; b Department of Medical Laboratory Science, College of Medicine and Health Sciences, Arba Minch University, Arba Minch, Ethiopia; c Department of Medical Laboratory Science, College of Health Sciences, Kebri Dehar University, Kebri Dehar, Ethiopia; d Department of Hematology and Immunohematology, School of Biomedical and Laboratory Science, College of Medicine and Health Sciences, University of Gondar, Gondar, Ethiopia.

**Keywords:** associated factors, Ethiopia, Harar, type 2 diabetes mellitus

## Abstract

Type 2 diabetes mellitus (T2DM) is the most common type of diabetes, accounting for over 90% of all diabetes cases worldwide. T2DM is usually developed in older ages and is usually associated with obesity, a sedentary lifestyle, or a high-calorie diet. This study aimed to determine the prevalence of T2DM and its associated factors among adult patients attending Outpatient Department at Jinella Health Center, Harar, Ethiopia. A facility-based cross-sectional study was conducted from July to August 2023. Face-to-face interview using a semi-structured questionnaire was used to collect socio-demographic and behavioral characteristics from 372 eligible participants. The random blood glucose level of the participants was measured from the capillary blood by pricking their fingers. Descriptive statistics was used to summarize the characteristics of the study participants. To determine factors associated with T2DM, bivariable and multivariable logistic regression analyses were used. The odd ratio with a 95% confidence interval was used to determine the strength of association between the predictor and T2DM. A *P*-value of <.05 in multivariable logistic analysis was considered statistically significant. The overall prevalence of T2DM was 15.3% (95% CI: 11.82–19.39%). The highest prevalence of T2DM was seen in female patients (61.4%). Furthermore, the prevalence was higher in the age group of 58 to 67 (36.8%). Being overweight (adjusted odds ratio (AOR) = 5.522; 95% CI: 2.643–11.534) and family history of diabetes mellitus (AOR = 0.114; 95% CI: 0.052–0.252) were significantly associated with T2DM. T2DM is one of the public health concerns in the adult population. Being overweight and family history of DM can increase the risk of developing T2DM. Relevant departments should establish effective policies and interventions to implement educational programs. Additionally, individuals should embrace a healthy lifestyle, increase their physical activity, and maintain a healthy weight. These measures can aid in type 2 diabetes management in the region.

## 1. Introduction

Diabetes mellitus (DM), is a chronic disorder that is manifested by elevated blood glucose levels or hyperglycemia. The hyperglycemia condition arises as a result of failure in insulin hormone production, action, or both.^[1]^ The pancreas is responsible for the production of this important hormone which enables circulatory glucose to reach the body’s cells, where it might be stored or transformed into energy.^[2]^ Furthermore, the metabolism of fat and protein depends on insulin.^[3]^

Etiologically, DM is classified into 2 types: type 1 diabetes mellitus (T1DM) and type type 2 diabetes mellitus (T2DM). T1DM is characterized by the destruction of insulin-producing β-cell of the islets of the pancreas by an autoimmune reaction which leads to a complete deficiency of insulin.^[2,4]^ On the other hand, hyperglycemia in T2DM arises from the inability of the body’s cells to respond fully to insulin, a condition termed insulin resistance.^[5]^ With the onset of insulin resistance, the hormone is less effective and, in due course, prompts an increase in insulin production. Over time, inadequate production of insulin can develop as a result of the failure of the pancreatic beta cells to keep up with demand.^[6]^

It has been noted that T2DM usually develops in older ages while T1DM usually affects children, though it can occur at any age.^[2,4]^ All age groups are affected by the emergence of T2DM, which poses a public health, financial, and social catastrophe.^[7]^ T2DM is usually associated with obesity, a sedentary lifestyle, or a high-calorie diet.^[8]^ Chronic calorie consumption can lead to changes in body composition and fat content, which harm glucose metabolism, including glucose oxidation and storage, insulin sensitivity, and insulin secretion.^[9]^ A sedentary lifestyle and low levels of physical exercise also contribute to a positive energy imbalance; the coinciding lower metabolic activity of skeletal muscle impacts both energy requirements and substrate oxidation, with consequences in equations for nutritional balance.^[10]^

Diabetes is a chronic health issue that is considered to be a public health concern.^[11]^ According to the International Diabetes Federation report, the global diabetes prevalence in 20 to 79-year-olds in 2021 was estimated to be 10.5% (536.6 million people), rising to 12.2% (783.2 million) in 2045. Diabetes prevalence was similar in men and women and was highest in those aged 75 to 79 years. Prevalence (in 2021) was estimated to be higher in urban (12.1%) than rural (8.3%) areas, and in high-income (11.1%) compared to low-income countries (5.5%). The greatest relative increase in the prevalence of diabetes between 2021 and 2045 is expected to occur in middle-income countries (21.1%) compared to high- (12.2%) and low-income (11.9%) countries.^[12]^

In sub-Saharan Africa, the number of people with impaired glucose tolerance is predicted to increase by 75.8%, from 26.9 million in 2010 to 47.3 million in 2030. This percentage is more than twice as high as the projected 37% worldwide rise.^[13]^ All age groups are affected by the emergence of T2DM in Ethiopia, which poses a public health, financial, and social catastrophe. According to a study conducted in Ethiopia, T2DM affects 6.5% of men and 6.6% of women population.^[7]^ Global diabetes-related health expenditures were estimated at 966 billion USD in 2021, and are projected to reach 1054 billion USD by 2045.^[12]^

Previously conducted studies didn’t address the socio-demographic and behavioral characteristics of the population that will expose to T2DM, particularly in the study area. Additionally, the earlier studies could not offer proof of the general prevalence of T2DM at Jinella Health Center (JHC) and did not fully consider all possible socio-demographic and behavioral factors. As a result, the objective of this Facility-based cross-sectional study was to determine the prevalence and associated factors of T2DM among adult patients attending the Outpatient Department (OPD) at JHC, Harar, Ethiopia.

## 2. Methods and materials

### 2.1. Study area and period

The study was conducted at JHC in Harar, Eastern Ethiopia, from July to August 2023. Harar is situated 511 km distant from the capital Addis Ababa. The region had a total population of 183,344 as of the 2007 national population census, of which 99,321 (54.2%) lived in urban areas and 84,023 (45.8%) in rural areas. About 62% of the population resides in the urban area including Harar. Estimates for the region’s population growth rates in urban and rural areas between 2007 and 2010 were 2.0% and 3.3%, respectively. The total population projection of the Harari regional state is estimated to be 276,000. From the total population, 62.6% live in urban, and the rest 37.4% live in rural areas. The living conditions in Harari are a blend of traditional and modern influences. Residents often engage in government work, agriculture, trade, and handicrafts, reflecting a lifestyle that values community and local resources. The majority of households had 4 to 6 family members. Regarding the wealth index, the majority of the population was found in the highest wealth index categories.^[14]^ According to the Harari Regional Health Bureau’s 2018 annual report, the region consists of 4 hospitals (two of which are public and private hospitals), and ten health centers.^[15]^ The JHC serves nearly 30,000 residents of the city of Harar in the Harari National Regional State, Ethiopia.^[16]^

### 2.2. Study design

A facility-based cross-sectional study design was used.

### 2.3. Source population

Patients attending OPD of JHC.

### 2.4. Study population

Patients attending OPD of JHC during the study period.

### 2.5. Sample size determination

#### 2.5.1. Sample size calculation and sampling technique

The sample size (n) was determined by using the single population proportion formula with the following assumptions. The proportion was taken from previously conducted research with a 31.5% prevalence of T2DM.^[17]^ Then it was calculated with a 95% confidence interval (Z = 1.96) and 5% marginal error (*d* = 0.05). The sample size is


$$\begin{array}{l} n=\frac{{\left({}^{Z\alpha }/{}_{2}\right)}^{2}\times p\times q}{d^{2}} \end{array}$$

where n = minimum sample size required for the study

${}^{Z\alpha }/{}_{2}=1.96$, (confidence interval)

*p* = proportion of the problem (0.315)

*q* = 1 − *P* = 1 − 0.315 = 0.685

*d* = margin of error (5%), 95% confidence interval

(1.96)^2^*0.315*0.685 **n = 338**

(0.05)^2^

Finally, by considering a 10%^[18]^ non-response rate the final sample size was determined as 372. The 372 study participants were enrolled in the study using a systemic random sampling technique.

### 2.6. Eligibility criteria

#### 2.6.1. Inclusion criteria

All adult patients who attended OPD of JHC and volunteered to participate were included in the study.

#### 2.6.2. Exclusion criteria

- Pregnant women
- Known DM patients

### 2.7. Study variables

#### 2.7.1. Dependent variable

- T2DM

#### 2.7.2. Independent variables

### 2.8. Socio-demographic pattern

- Age
- Sex
- Place of residency
- Educational status
- Occupation
- Marital status

Behavioral characteristics

- Physical exercise
- Alcohol consumption
- Cigarette smoking

Clinical characteristics

- Blood pressure
- Body mass index (BMI)
- Family history of DM

### 2.9. Operational definitions

- T2DM: Having a random blood glucose level of > 200 mg/dL^[19]^
- **Physical exercise:** is scheduled. Organized and planned physical activity that has an ultimate purpose.^[20]^
- **Regular smoker:** An adult who, in his or her lifetime, has smoked at least 100 cigarettes, and who now smokes every day.^[21]^
- **Normal blood pressure (BP):** Normal BP < 140/90 mm Hg^[22]^
- **BMI:** Underweight = <18.5

Normal = 18.5–24.9

Overweight = 25–29.9^[23]^

- A**lcohol consumption:** regular consumption of alcoholic drinks

### 2.10. Data collection procedure

#### 2.10.1. Socio-demographic characteristics and behavioral data collection

After obtaining informed written consent, a pre-tested semi-structured questionnaire was used to collect socio-demographic and behavioral data from patients. To check for respondents understanding the questions as intended, ensuring clarity and relevance, pretesting of the questionnaire was done at Jegol Hospital with 19 respondents. The results indicated that the questionnaire posed no issues in understanding and responding to questions, and all participants answered the questions without difficulty. The questionnaire was used to make face-to face interviews with the participants. Some of the socio-demographic data that were collected include age, sex, occupation, residence, and level of education. Behavioral data such as the habit of regular physical exercise, cigarette smoking, and history of alcohol intake were collected using questionnaires and face-to-face interviews.

#### 2.10.2. Random blood glucose measurement

The random blood glucose levels of the participants were measured from the capillary blood by pricking their fingers. After finger pricking, the first drop of blood was wiped out then the second drop of blood was used for determining the blood glucose level.

#### 2.10.3. Method of measuring blood pressure

During the measuring process, an inflating rubber bag is placed within a cuff that is wrapped around the subject’s arm and placed over the brachial artery. The cuff is inflated to a sufficient pressure to seal the artery. The thumb valve is then opened to discharge the air pressure. When the pressure within the cuff matches the pressure within the artery, the artery opens and blood starts to flow back into the occluded portion of the artery.

Pulse noises start as soon as the blood flows back into the vessel. A stethoscope placed over the brachial pulse point allows one to hear these sounds. As the cuff gradually deflates, the sounds last for a while until becoming inaudible.

Through tubing, the cuff is linked to a manometer that displays the arterial pressure. The manometer reads the systolic blood pressure at the moment the first pulse sounds. Diastolic blood pressure is the last sound to be heard. Rather than the sound disappearing, the diastolic blood pressure in children is commonly measured by the muffling of sound, or fourth sound.

#### 2.10.4. Body mass index measurement

BMI is a measure of weight adjusted for height, calculated as weight in kilograms divided by the square of height in meters (kg/m^2^).

#### 2.10.5. Data quality control measurement

A Standard operation procedure was followed for every procedure that was performed in this study. A capillary blood sample was used other than venous blood for the measurement of blood glucose level. The questionnaire was prepared in English and then translated into Amharic, Afanoromo, and Harari by the principal investigator to check for consistency. A training was given to data collectors about the objective and relevance of the study, confidentiality issues, study participants’ rights, consent issues, techniques of interview, and proper recording of results on the record sheet prepared for the research purpose. Also, laboratory technologists were oriented on the objective and relevance of the study, registration of laboratory test results, and laboratory test result confidentiality. The investigator followed and frequently checked every process to ensure the completeness and consistency of the collected data. Finally, all the results were checked for mislabeling and completeness and recorded appropriately on the registration worksheet daily and then transferred to registration books and a computer.

#### 2.10.6. Data processing and analyzing

Data was entered in Epi info version 7.2 and transferred to SPSS version 22 for analysis. Descriptive statistics like frequencies, tables, and imported figures were used to summarize the characteristics of the study population. To determine the prevalence and associated factors of T2DM among adult patients, logistic regression analysis was used. The odds ratio with its 95% interval was used to determine the strength of association between the predictor and dependent variable. Variables whose level is statistically significant (*P* < .25) on bivariable analysis were entered jointly into a multi-variable logistic regression analysis. A *P*-value of <.05 in multi-variable logistic regression analysis was considered statistically significant.

### 2.11. Ethical considerations

The study was conducted after it was ethically approved by the ethical clearance committee of the Department of Medical Laboratory Sciences, Institute of Health Sciences, Jigjiga University (JJU/CMHS/MLS/078/15). A permission letter was obtained from JHC. Informed written consent was obtained from the patients and the findings were kept confidential. In case of abnormal results, it was informed to their medical doctors to get adequate treatment.

## 3. Result

### 3.1. Socio-demographic characteristics of adult T2DM patients

A total of 372 study participants were enrolled in this study, with 54.3% (202/372) of them being Female and 76.6% (285/372) residing in urban. The study participants’ mean age was 47 years with a range of 18 to 90 years. The majority of the study participants, 19.9% (74/372) and 51.9% (193/372) were 58 to 67 years old and married, respectively (Table 1).

**Table 1:** Socio-demographic characteristics of adult type 2 diabetes mellitus patients.

Socio-demographic characteristics	Frequency	Percentage
Sex		
Male	170	45.7
Female	202	54.3
Age		
18–27	48	12.9
28–37	67	18.0
38–47	70	18.8
48–57	68	18.3
58–67	74	19.9
≥68	45	12.1
Place of residence		
Urban	285	76.6
Rural	87	23.4
Educational status		
Unable to read and write	62	16.7
Able to read and write	106	28.5
Elementary	26	7.0
High school	56	15.0
College and above	122	32.8
Occupation		
Student	41	11.0
Governmental	95	25.5
Private	88	23.7
Housewife	77	20.7
Other	71	19.1
Marital status		
Single	88	23.7
Married	193	51.9
Divorced	51	13.6
Widowed	40	10.8

### 3.2. Clinical and behavioral characteristics of adult T2DM patients

About 17.2% (64/372) of the study participants had a family history of DM. From the study participants, 27.2% (101/372) were cigarette smokers. The number of patients with hypertension was 21.5% (80/372). About 14% (52/372) perform regular physical exercise and 69.6% (259/372) has a normal body weight (Table 2).

**Table 2:** Clinical and behavioral characteristics of adult type 2 diabetes mellitus patients.

Clinical and behavioral characteristics	Frequency	Percentage
Smoking cigarette		
Yes	101	27.2
No	271	72.8
Drinking alcohol		
Yes	95	25.5
No	277	74.5
Physical exercise		
Yes	52	14.0
No	320	86.0
BMI		
Underweight	5	1.3
Normal weight	259	69.6
Over weight	108	29.1
Presence of hypertension		
Yes	80	21.5
No	292	78.5
Family history of DM		
Yes	64	17.2
No	308	82.8
No	327	87.9

DM = diabetes mellitus.

### 3.3. Prevalence of T2DM among adult patients at Jinella Health Center

**Figure 1.**
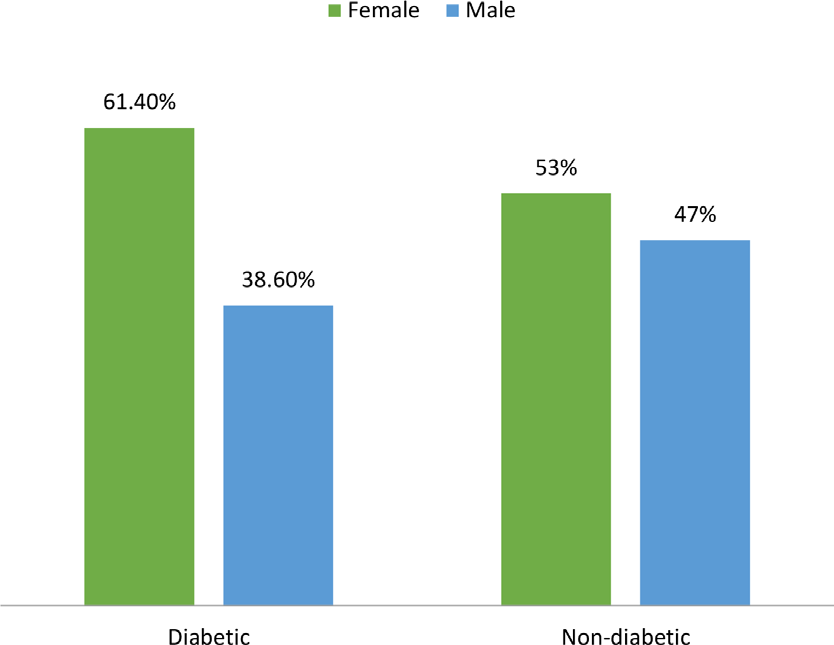
Prevalence of type 2 diabetes mellitus among adult patients attending OPD at JHC, Harar, Ethiopia. JHC = Jinella Health Center, OPD = Outpatient Department.

The overall prevalence of T2DM was 15.3% (57/372), (95% CI: 11.82–19.39%). Out of this abnormality, females had a higher prevalence of T2DM (61.4%) than males did. The highest prevalence was observed in the age group of 58 to 67 with 36.8%. Moreover, 71.9% of urban residents had T2DM compared to the rural population. Furthermore, a high prevalence of T2DM was also seen in overweight patients with 63.2% (Fig. 1).

### 3.4. Factors associated with type 2 diabetic mellitus

Bivariable and multivariable logistic regression analyses have been performed. In bivariable logistic regression analysis, sex (crude odds ratio (COR) = 0.709; 95% CI: 0.338–1.263), age, occupation (COR = 0.482; 95% CI: 0.146–1.592), smoking cigarette (COR = 1.878; 95% CI:1.040–3.391), alcohol consumption (COR = 2.283; 95% CI: 1.264–4.122), performing physical exercise (COR = 0.420; 95% CI: 0.145–1.214), BMI (COR = 0.176; 95% CI: 0.097–0.321), and family history of DM (COR = 6.762; 95% CI: 3.627–12.606) showed association with T2DM. Consequently, these variables were subjected to multivariable binary logistic regression. On the other hand, the study participants’ residence, educational status, marital status, and hypertension did not show any statistical association with T2DM.

However, in multivariable analysis, being overweight (adjusted odds ratio (AOR) = 5.522; 95% CI: 2.643–11.534) and family history of DM (AOR = 0.114; 95% CI: 0.052–0.252) were significantly associated with T2DM (Table 3).

**Table 3:** Factors associated with type 2 diabetes mellitus.

Characteristics	Category	Diabetic mellitus		COR (95% CI)	*P*-value	AOR (95% CI)	*P*-value
		Diabetic	Non-diabetic				
		N (%)	N (%)				
Sex	Male	22 (38.6)	148 (47)	1	–	–	–
	Female	35 (61.4)	167 (53)	0.71 (0.40–1.26)	.24	1.10 (0.42–2.86)	.85
Age	18–27	5 (8.8)	43 (13.7)	1	–	–	–
	28–37	9 (15.8)	58 (18.4)	0.47 (0.14–1.51)	.20	0.65 (0.08–5.11)	.67
	38–47	8 (14)	62 (19.7)	0.62 (0.23–1.71)	.37	0.98 (0.22–4.32)	.98
	48–57	5 (8.8)	63 (20)	0.52 (0.18–1.46)	.21	0.29 (0.07–1.21)	.09
	58–67	21 (36.8)	53 (16.8)	0.32 (0.10–1.02)	.05	0.26 (0.06–1.16)	.08
	≥68	9 (15.8)	36 (11.4)	1.59 (0.65–3.85)	.31	1.75 (0.54–5.70)	.35
Residency	Rural	16 (28.1)	71 (22.5)	1	–	–	–
	Urban	41 (71.9)	244 (77.5)	0.75 (0.34–1.41)	.37	–	–
Educational status	College and above	20 (35.1)	102 (32.4)	1	–	–	–
	Unable to read and write	11 (19.3)	51 (16.2)	0.78 (0.37–1.63)	.50	–	–
	Able to read and write	14 (24.6)	92 (29.2)	1.10 (0.49–2.47)	.82	–	–
	Elementary	6 (10.5)	20 (6.3)	1.53 (0.55–4.29)	.42	–	–
	High school	6 (10.5)	50 (15.9)	0.61 (0.23–1.62)	.32	–	–
Occupational status	Student	4 (7)	37 (11.7)	1	–	–	–
	Governmental	13 (22.8)	82 (26.0)	0.48 (0.15–1.59)	.23	0.83 (0.09–8.06)	.88
	Private	14 (24.6)	74 (23.5)	0.71 (0.31–1.64)	.42	0.67 (0.19–2.31)	.52
	Housewife	13 (22.8)	64 (20.4)	0.84 (0.37–1.94)	.69	0.91 (0.27–3.12)	.89
	Other	13 (22.8)	58 (18.4)	0.91 (0.39–2.11)	.82	0.78 (0.22–2.76)	.70
Marital status	Single	9 (15.8)	79 (25.1)	1	–	–	–
	Married	27 (47.4)	166 (52.7)	0.54 (0.19–1.56)	.25	–	–
	Divorced	14 (24.6)	37 (11.7)	0.77 (0.31–1.91)	.57	–	–
	Widowed	7 (12.3)	33 (10.5)	1.78 (0.64–4.95)	.27	–	–
Smoking cigarette	No	35 (61.4)	236 (74.9)	1	–	–	–
	Yes	22 (38.6)	79 (25.1)	1.88 (1.04–3.39)	.04	0.63 (0.24–1.61)	.33
Drinking alcohol	No	34 (59.6)	243 (77.1)	1	–	–	–
	Yes	23 (40.4)	72 (22.9)	2.28 (1.26–4.12)	.01	0.78 (0.36–1.67)	.52
Performing physical exercise	Yes	4 (7.0)	48 (15.2)	1	–	–	–
	No	53 (93.0)	267 (84.8)	0.42 (0.14–1.21)	.11	1.06 (0.27–4.09)	.94
BMI	Normal weight	21 (36.8)	238 (75.5)	1	–	–	–
	Under weight	0 (0.00)	5 (1.6)	0.01 (0.01)	1.00	–	1.00
	Over weight	36 (63.2)	72 (22.9)	0.18 (0.10–0.32)	.01	5.52 (2.64–11.53)	.01
Presence of hypertension	No	26 (45.6)	266 (84.5)	1	–	–	–
	Yes	31 (54.4)	49 (15.5)	6.61 (3.61–12.10)	1.00	–	–
Presence of family history of DM	No	30 (52.6)	278 (88.3)	1	–	–	–
	Yes	27 (47.4)	37 (11.7)	6.76 (3.63–12.61)	.01	0.11 (0.05–0.25)	.01
	Yes	12 (21.1)	33 (10.5)	2.28 (1.10–4.74)	.03	0.26 (0.10–0.65)	.01

AOR = adjusted odds ratio, BMI = body mass index, COR = crude odds ratio, DM = diabetes mellitus.

## 4. Discussion

In emerging nations like Ethiopia, non-communicable diseases are increasingly bearing a double weight of public health issues. In addition, developing countries are seeing an increase in the prevalence of type 2 diabetes. This study aimed to determine the prevalence and associated factors of T2DM among patients attending OPD of JHC, Harar, Ethiopia. In this study, the overall prevalence of T2DM was 15.3 % (95% CI: 11.82–19.39%). This high prevalence of T2DM might be related to rapid urbanization, lifestyle changes, and limited resources.^[24]^ This finding is in line with a cross-sectional study conducted in Uganda (18.7%),^[25]^ Thailand (17.7%),^[26]^ and China (14.3)%.^[27]^

On the other hand, the prevalence of T2DM in this study was higher compared to a study conducted in Northern India (8.3%),^[28]^ Russia (5.4%),^[29]^ and Harar, Ethiopia (7.1%).^[30]^ This variation could result from variations in the sample sizes, research designs used, participant socioeconomic characteristics, and the type of glucose measurement techniques used in the studies. The statistical power and generalizability while calculating the sample size might lead to this variation. Moreover, study design can also contribute to this variation following the variation in methodology and bias and confounding factor handling. Variations in socioeconomic characteristics of the populations can also impact the variation by influencing health disparities and access to resources like health care, nutrition, and lifestyle. The glucose measurement technique, and reference range used can also contribute to this variation. However, the prevalence of T2DM in this study was lower than a study conducted in Jigjiga, Ethiopia that reported an elevated blood glucose level in 31.5% of the study participants.^[17]^

This study also assessed factors associated with T2DM. The multivariable logistic analysis revealed that being overweight (AOR = 5.522; 95% CI: 2.643–11.534) and family history of DM (AOR = 0.114; 95% CI: 0.052–0.252) were significantly associated with T2DM. In this study, overweight patients were 5 times at risk of developing T2DM compared to normal-weight patients.

The pathophysiology connecting obesity and diabetes is chiefly attributed to 2 factors: insulin resistance and insulin deficiency. Obesity causes sustained elevation in plasma-free fatty acids levels, both in the basal state and following glucose load which present a major factor for insulin resistance. Clinical studies in healthy volunteers with acute elevation of plasma-free fatty acids resulted in whole-body insulin resistance.^[31,32]^ This finding is in line with a study conducted in Mongolia,^[33]^ and Ethiopia.^[17]^ Furthermore, a family history of DM was also significantly associated with T2DM. This finding is in agreement with a study conducted by Endris T.,^[18]^ Fiseha T.,^[34]^ and Tesfaye T.,^[35]^ in Ethiopia.

## 5. Conclusions and recommendations

T2DM is one of the public health concerns in the adult population. Being overweight and family history of DM can increase the risk of developing T2DM. Relevant departments should establish effective policies and interventions to implement educational programs. Additionally, individuals should embrace a healthy lifestyle, increase their physical activity, and maintain a healthy weight. These measures can aid in type 2 diabetes management in the region.

## 6. Strengths and limitations of the study

The study’s strengths include the identification of the prevalence and associated factors of T2DM in OPD patients at JHC. The first major limitation of this study was being cross-sectional nature which does not reveal causal relations between independent variables and the prevalence of T2DM. The other limitation of this study is the use of only Rando Blood to diagnose DM, which may underestimate the prevalence of DM.

## Acknowledgments

The authors would like to thank Jigjiga University, the Institute of Health Sciences, Department of Medical Laboratory Science for their support in conducting this study. Moreover, the authors are grateful to the health center staff and study participants for all their support.

## Author contributions

**Conceptualization:** Fasil Getu, Abdikani Rashid, Muluken Walle.

**Data curation:** Fasil Getu, Abdikani Rashid, Muluken Walle.

**Formal analysis:** Fasil Getu, Addisu Tesfaye.

**Funding acquisition:** Fasil Getu, Addisu Tesfaye, Muluken Walle.

**Investigation:** Fasil Getu, Muluken Walle.

**Methodology:** Fasil Getu, Addisu Tesfaye, Birhanu Genanew, Muluken Walle.

**Project administration:** Fasil Getu, Abdikani Rashid.

**Resources:** Fasil Getu, Addisu Tesfaye, Birhanu Genanew.

**Software:** Fasil Getu, Muluken Walle.

**Supervision:** Fasil Getu, Abdikani Rashid, Muluken Walle.

**Validation:** Fasil Getu, Birhanu Genanew, Abdikani Rashid.

**Visualization:** Fasil Getu.

**Writing – original draft:** Fasil Getu, Birhanu Genanew, Muluken Walle.

**Writing – review & editing:** Fasil Getu, Muluken Walle.
